# Development of a Self-Powered Piezo-Resistive Smart Insole Equipped with Low-Power BLE Connectivity for Remote Gait Monitoring

**DOI:** 10.3390/s21134539

**Published:** 2021-07-01

**Authors:** Roberto de Fazio, Elisa Perrone, Ramiro Velázquez, Massimo De Vittorio, Paolo Visconti

**Affiliations:** 1 Department of Innovation Engineering, University of Salento, 73100 Lecce, Italy; roberto.defazio@unisalento.it (R.d.F.); elisa.perrone5@studenti.unisalento.it (E.P.); massimo.devittorio@unisalento.it (M.D.V.); 2 Facultad de Ingeniería, Universidad Panamericana, Aguascalientes 20290, Mexico; rvelazquez@up.edu.mx; 3 Center for Biomolecular Nanotechnologies, Italian Technology Institute IIT, 73010 Arnesano, Italy

**Keywords:** smart insole, health monitoring, plantar pressure distribution, piezoresistivity, BLE

## Abstract

The evolution of low power electronics and the availability of new smart materials are opening new frontiers to develop wearable systems for medical applications, lifestyle monitoring, and performance detection. This paper presents the development and realization of a novel smart insole for monitoring the plantar pressure distribution and gait parameters; indeed, it includes a piezoresistive sensing matrix based on a Velostat layer for transducing applied pressure into an electric signal. At first, an accurate and complete characterization of Velostat-based pressure sensors is reported as a function of sizes, support material, and pressure trend. The realization and testing of a low-cost and reliable piezoresistive sensing matrix based on a sandwich structure are discussed. This last is interfaced with a low power conditioning and processing section based on an Arduino Lilypad board and an analog multiplexer for acquiring the pressure data. The insole includes a 3-axis capacitive accelerometer for detecting the gait parameters (swing time and stance phase time) featuring the walking. A Bluetooth Low Energy (BLE) 5.0 module is included for transmitting in real-time the acquired data toward a PC, tablet or smartphone, for displaying and processing them using a custom Processing^®^ application. Moreover, the smart insole is equipped with a piezoelectric harvesting section for scavenging energy from walking. The onfield tests indicate that for a walking speed higher than 1 ms^−1^, the device’s power requirements (i.e., $\bar{P}=5.84\;\mathrm{mW}$) was fulfilled. However, more than 9 days of autonomy are guaranteed by the integrated 380-mAh Lipo battery in the total absence of energy contributions from the harvesting section.

## 1. Introduction

Today technology is increasingly in close contact with humanity, an inseparable bond that promises, used with intellect and parsimony, to improve our lives in every aspect [1,2,3]. Thanks to the Internet of Things (IoT), these technologies allow us to be connected and keep under control every aspect of our life, from the home to the car, to health monitoring [4,5,6]. The latter application is significant, both for controlling chronic diseases and the vital parameters and performances of sportsmen; the recent advances in low power electronics and communications have enabled the development of IoMT (Internet of Medical Things) or IoHT (Internet of Healthcare Things) devices with considerable technological improvements in the healthcare and culture of prevention. Specifically, the wearable device market is growing rapidly ensuring prompt intervention, punctual prevention, and time-saving [7,8,9]. Recent technological advances have led to the development of biomedical wearable devices, for instance, for gait analysis, which is crucial as plantar pressure distribution reflects the foot structure and user posture [10,11]. These smart insoles are based on pressure sensors, such as force-sensitive resistors (FSRs), to collect data related to plantar pressure. This paper reports on the design of a low-cost, compact, and reliable smart insole for monitoring the plantar pressure distribution and gait parameters. It is equipped with a matrix of piezoresistive sensors based on the Velostat^®^ layer (manufactured by 3M Electronics division, Saint Paul, MN, USA), based on a sandwich structure [12,13]. The main contributions of the scientific work are:

- A comprehensive characterization of Velostat^®^-based piezoresistive sensors with different sizes, support materials, fixing methods, and pressure trends to determine the most suitable solution for implementing the sensing matrix;
- Design of the sensing matrix, including 8 FSRs with size 3 × 1 cm^2^, interfaced with a conditioning and acquisition section based on Arduino Lilypad board;
- Testing of the low-power smart insole by acquiring pressure and acceleration data provided by the sensing matrix and 3-axis accelerometer; the insole includes a piezoelectric harvesting section to scavenge energy from the user walking [8,14];
- Development of a custom Processing^®^ application, implementing an interpolation method for extending the acquired pressure map.

The Arduino Lilypad board acquires and processes the sensors’ data, optimizes the power consumption, and manages the data transmission, using a Bluetooth Low Energy (BLE) module, toward a host device (e.g., PC, tablet or smartphone) for the post-processing. By exploiting the low power modalities of used components, the insole power requirement has been minimized to only 5.84 mW. The onfield tests demonstrated that, for walking speed higher than 1 ms^−1^, the harvesting section entirely covered the device power requirement (i.e., $\bar{P}=5.84\;\mathrm{mW}$). Nevertheless, the LiPo battery ensures an autonomy of 9 days in the absence of energy contribution from the harvesting section.

The article is arranged as follows: in the following section, an overview of smart insoles based on FSRs and inertial sensors is presented; in Section 3, the setup employed to characterize Velostat-based piezoresistive sensors is described. Then, the characterization of realized Velostat-based sensors is reported as a function of size, support material, fixing method, and pressure trend (Section 4). Moreover, the design of the sensing matrix based on a sandwich structure is introduced, integrated with a low power acquisition and processing section. Subsequently, the Processing^®^ application to post-process and display the data received by the smart insole is presented. Finally, in Section 5, the obtained results are discussed.

## 2. An Overview of Smart Insoles for Plantar Pressure Detection and Gait Analysis

Recent advances in the biomedical field have led to the development of new advanced wearable devices, including those intended for gait analysis [15,16]. The most widespread applications regard the monitoring of diabetes ulcers [17], the evaluation of sports performance [18], workers’ conditions [19], and biometric recognition [20]. The assessment of plantar pressure distribution is essential to understand the lower limbs’ functioning, design footwear, and prevent injuries [21]. In recent years, the design of smart insoles, equipped with sensors for monitoring plantar pressure, has attracted considerable interest. IoT technologies play a fundamental role in developing these devices, enabling capillary, accurate, and continuous tracking of user conditions [22]. To detect the pressure distribution, piezoresistive force sensors (FSRs) are used instead of the piezoelectric, capacitive, and optical sensors [23]. The FSRs are usually featured by a linear trend of conductivity with the applied force [24]. Soft materials are preferred because featured by high sensitivity, lightweight, low invasiveness, and a long lifetime [25]. These features make FSRs suitable for realizing wearable devices adopting proper strategies for improving sensitivity and flexibility [26]. A configuration to detect the resistance variations is to configure the sensor as a voltage divider, acquiring the non-linear voltage on the sensor and managing the non-linearity using software methods. Another applied approach uses a trans-impedance amplifier: its output voltage varies linearly with the current through the sensor and thus with the applied force. To obtain the complete plantar distribution on the insole, it is necessary to use multiple FSRs positioned at the toe, metatarsal, and heel, corresponding to bony prominences where high pressures are often applied. In [27], the authors identified 15 interest areas on the sole foot, supporting most of the bodyweight and regulated by the body balance. Therefore, to acquire all the signals, it is needed a multiplexing scheme for the FSRs to use then only one analog channel [28].

Human walking represents an energy source exploitable for feeding wearable devices, such as smart insoles and socks [29]. According to the transduction mechanism, several solutions were proposed in the literature exploiting electromagnetic induction, piezoelectric, and triboelectric effects for scavenging energy from body vibrations, body inertia, and foot pressure [30,31]. Besides, several efforts have been made to improve efficiency, wearability, and durability and to reduce the cost of harvesting solutions, opening to the development of new self-powered wearable devices [32,33].

**Figure 1:**
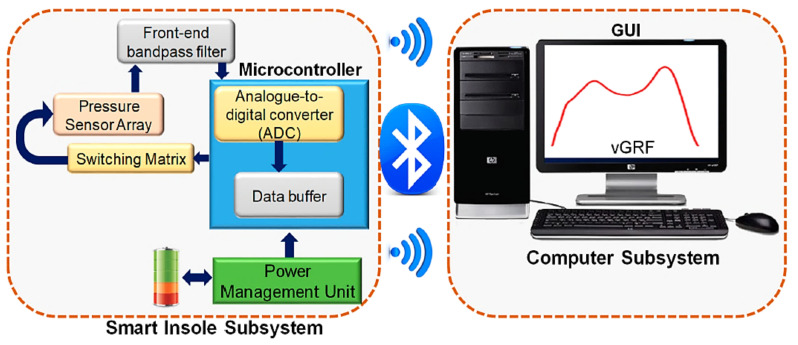
Overall block diagram of the insole data acquisition system, Adapted from Ref. [34].

In [34], the authors proposed a low-cost characterization system to calibrate FSRs, ceramic and flexible piezoelectric sensors, and characterize the designed insoles. To evaluate the user walking mode, the authors acquired and time-segmented the vGRF (ground reaction forces) signal to get its mean value and standard deviation for a gait cycle. The results showed that the FSR is the most effective one for insole applications; instead, piezoelectric sensors can be used only for detecting the start and end of the cycle. The data acquisition system, whose block diagram is shown in Figure 1, collected sensors data and wirelessly sent them to a host computer for post-processing and visualization.

**Figure 2:**
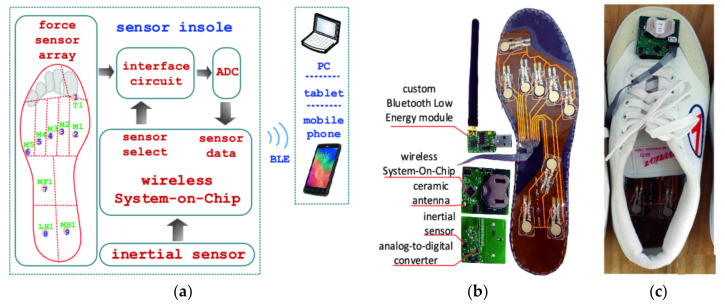
Smart insole proposed in [35], Copyright Clearance Center_Springer Nature_Copyright Permission_Figure 2 and Figure 3.pdf: block diagram of the proposed solution (**a**); view of the prototype with highlighted main sections (**b**); insole installed in a shoe (**c**).

**Figure 3:**
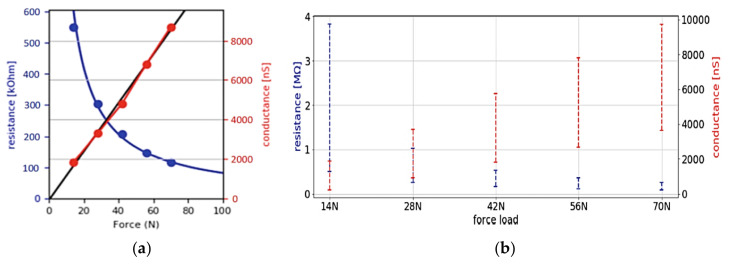
Characteristics of force sensors (**a**); comparison of the force–conductance ranges during calibration of force sensors (**b**) [35], ® by Copyright Clearance Center_Springer Nature_Copyright Permission_Figure 2 and Figure 3.pdf.

Another configuration for realizing smart insole is illustrated in Figure 2 [35]. The authors produced six pairs of insoles to cover the most common foot sizes, using nine circular FlexiForce sensors, located respectively under the toe (T1), metatarsals (M1–M5), middle part (MF7), and heel (LH1, MH1), as illustrated in Figure 2a [36]. A multiplexing scheme was used to acquire the sensor signals and then digitize them using an external 12-bit analog-to-digital converter (ADC). Their study demonstrated the importance of FSRs calibration; specifically, the characteristic of the sensors belonging to the same batch (Figure 3a) could differ significantly between the different realized insoles (Figure 3b).

**Figure 4:**
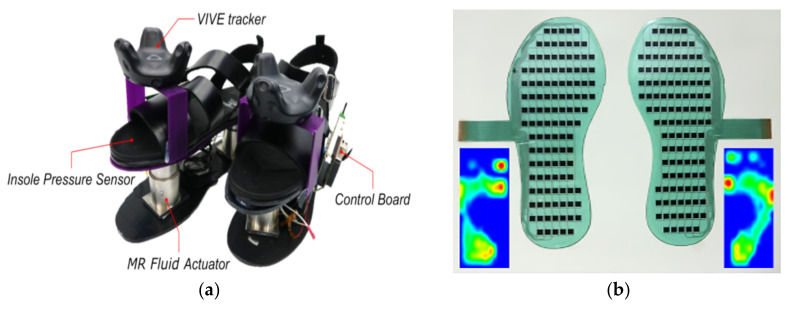
RealWalk haptic shoes (**a**); Sensor matrix (**b**) Adapted from [37].

Sang-Youn Kim et al., in [37], created a pair of tactile shoes (Figure 4) that recreate the realistic sensations of walking on different terrains. They were engaged in walk-in virtual reality, employing pressure sensors on the insole, foot position tracker, haptic rendering for multi-modal interaction, and MR (MagnetoRheological) fluid actuators. Specifically, the MR actuators adjusted the MR fluid’s viscosity by varying the magnetic field intensity according to the selected virtual ground surface.

**Figure 5:**
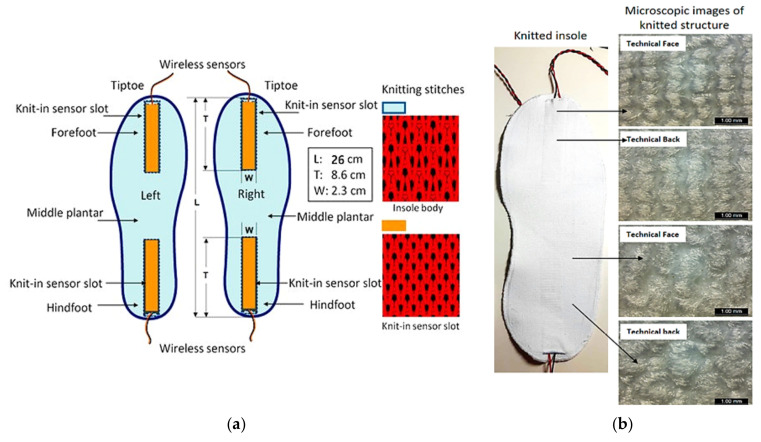
Schematic diagram of the self-powered intelligent insole detailing the fabric’s knitted motif (**a**) and the related prototype (**b**) Adapted from [38].

Wei Wang et al. presented self-powered insoles integrated with piezoelectric poly vinylidene fluoride (PVDF) nanogenerators (NGs) and manufactured using 3D flatbed knitwear without seams for monitoring user gait and scavenging the walking energy [38]. The NGs were made by growing aluminum electrodes on the PVDF film. The characterization results for different forefoot, heel positions, and walking speeds provided a maximum open-circuit voltage of 41 V for a 168.1 μW power (Figure 5a).

**Figure 6:**
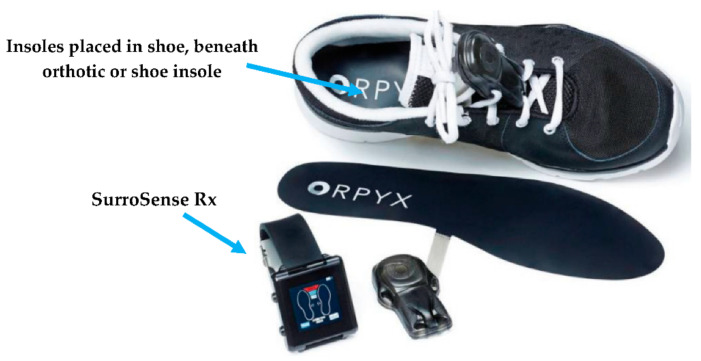
“SurroSense Rx” smart insole for curing plantar pressure during daily activities, Adapted from [39].

Footwear plays an essential role in foot health, especially for patients with diabetes, to prevent and treat diabetic foot ulceration. In [39], the authors reported on a health device to assess plantar pressures and provide feedback based on set pressure thresholds. The developed insole, called “SurroSense Rx”, stimulated plantar pressure during daily activities (Figure 6). Each insole was equipped with eight pressure sensors located in the highest risk regions of plantar ulcers and 6-DOF (degree of freedom) force/torque sensors; clinical results showed a reduction of ulcer recurrence rate by more than 50%.

**Figure 7:**
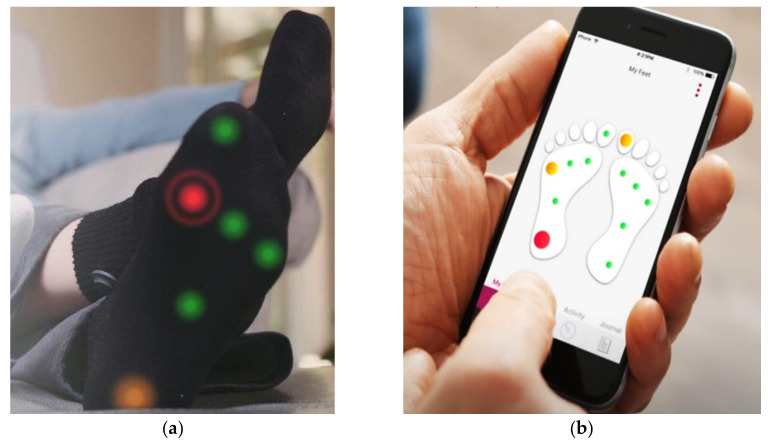
Smart socks developed by Siren with a detection system that can help to detect the first signs of foot ulcers: sensitive socks (**a**) and smartphone app (**b**), Adapted from [41].

D. Aggarwal et al. proposed novel smart socks allowing physiotherapists to remotely follow patients status [40]; the socks included three pressure and movement sensors for collecting data on weight distribution, movement freedom, and feet orientation, to be shared in real-time, via a web platform, with the physiotherapist. The results indicated that the smart socks were very helpful to understand better the patient’s body conditions and decide the most suitable therapy. Similarly, the Siren startup presented novel smart socks offering constant measurements of gait parameters [41]. The proposed socks monitor the feet health of diabetics, thanks to temperature sensors intertwined with the sock fabric, to detect temperature increase due to inflammation (Figure 7a). The information is transmitted to the smartphone app, which immediately alerts the user with a notification or SMS (Figure 7b). In addition, the socks can integrate various sensors, such as humidity, pressure, light and movement sensors, LEDs, and RFID chips.

Bonbouton Co. patented a smart insole to monitor the foot health of diabetic patients, detecting the skin temperature, pressure, and other data using a graphene-based sensor [42]. The collected data were sent to a custom app, accessible by both the patient and doctor, who can determine whether an intervention or a treatment is required.

**Figure 8:**
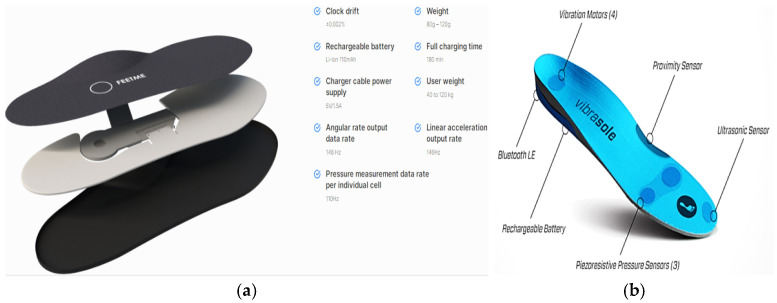
Top View of the FeetMe [43] (**a**) and Vibrasole insoles, Adapted from [44] (**b**).

**Figure 9:**
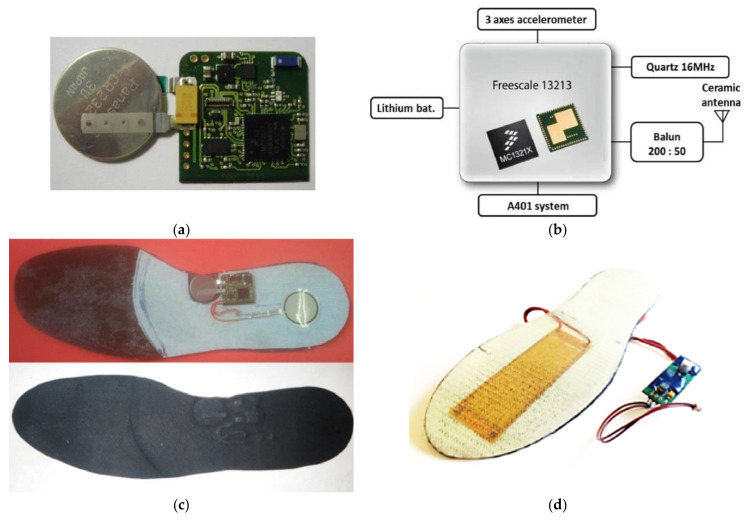
Prototypes of smart insole proposed in [45], Copyright 2018 Elsevier; first prototype (**a**) block diagram of the first device version (**b**); prototype of the smart insole (**c**), and the second version of the insole with the energy harvesting system (**d**).

Other similar devices are the FeetMe insoles for performing gait analysis or monitoring user parameters (Figure 8a) [43]. Vibrasole is an intelligent insole, based on FSRs, for helping elderly people to monitor and manage their balance while walking and standing [44]. The Vibrasole aids diabetic users that suffer from feet ulcers with low random vibrations (Figure 8b). Inertial sensors are also widely exploited to monitor the gait parameters quickly, reliably, and non-invasively. In particular, Y. Charlon et al. presented a smart insole able to detect step count, covered distance, and gait speed [45]. Two prototypes were developed and tested in real use conditions; the first one included an MC13213 microcontroller to acquire data from ADXL345 accelerometer and A401 piezoresistive sensor (Figure 9a). Moreover, the device was equipped with a radio beacon to send acquired data to a PC, which stored them in a remote database, accessible by a web application (Figure 9b). To power supply the device, a 135-mAh lithium battery was used. The second prototype (Figure 9c) included an onboard energy harvesting system with an MFC 13213 piezoelectric generator (Figure 9d). They demonstrated that the harvesting section could scavenge 0.3 mW for 1 m/s walking speed. Therefore, for frail elderly patients, who walks at an average speed of 1 m/s, only 3/10 of the energy requirement was fulfilled (1 mW); an additional lithium battery was added to provide the required energy.

In [46], the developed smart insole was constituted by three subsystems; the first one was equipped with an array of 48 pressure sensors to obtain a plantar pressure map, a 3-axis accelerometer, a 3-axis gyroscope, and a 3-axis compass to measure the user movement. The second subsystem was the signal acquisition and transmission module, including a 12-bit ADC operating with a sample rate up to 100 Hz. The third subsystem was represented by sensors collection and a processing module. The data were transmitted via Bluetooth to a smartphone and, in turn, shared with centralized servers to monitor the user gait in real-time. In [47], an insole was proposed to assess long-term chronic diseases affecting older people, such as stroke, dementia, Parkinson’s disease, cancer, heart disease, and diabetes. It included four sections; the first consists of 31 piezo-sensors connected to a 12 bit ADC and a microcontroller to read the pressure data at a 5 Hz sample rate. The second subsystem was equipped with the MPU6000 IMU to gather information about the wearer’s movement and position. The third section was the power management system; a 280-mAh Li-ion battery fed the device, allowing 120 min of continuous operation. The fourth subsystem was the communication section enabling the data transmission with a smartphone or other devices within a 3-m radius.

Table 1 summarizes the scientific works previously analyzed and discussed from the point of view of typology and number of used pressure sensors, the availability of wireless connectivity, and acquired parameters. Pedar X^®^ insoles are featured by the highest number of pressure sensors, providing a high degree of detail for detecting the plantar pressure map both in static and dynamic conditions, given the high acquisition rate (100 Hz). However, the device structure seems quite inconvenient and invasive compared to the other solutions discussed, resulting in poor applicability in daily life.

## 3. Materials and Methods

### 3.1. Technical Specifications and Previous Characterizations of the Piezoresistive Layer

**Figure 10:**
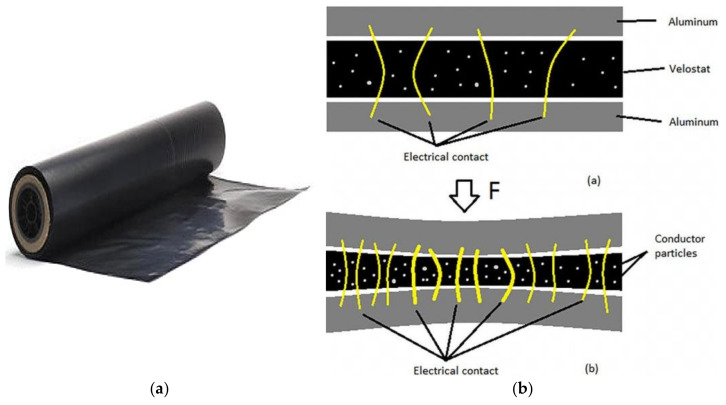
Pressure-sensitive conductive sheet of piezoresistive Velostat film (**a**) and related internal structure (**b**), Adapted from [50].

The Velostat piezoresistive material, also known as Linqstat, was developed by Custom Materials, now part of the 3M company and was later purchased by “Desco Industries” in 2015, becoming a US brand (Figure 10a) [13].

It is a packaging material constituted by a polymeric layer (polyolefins) impregnated with carbon powder to make it electrically conductive. Its main feature is to change its electric resistance with bending or pressure due to the changing of the geometric parameters (Figure 10b). Since the layer’s resistance decreases when pressure is applied, this reading can indicate when the weight is applied or removed from the sensor.

**Figure 11:**
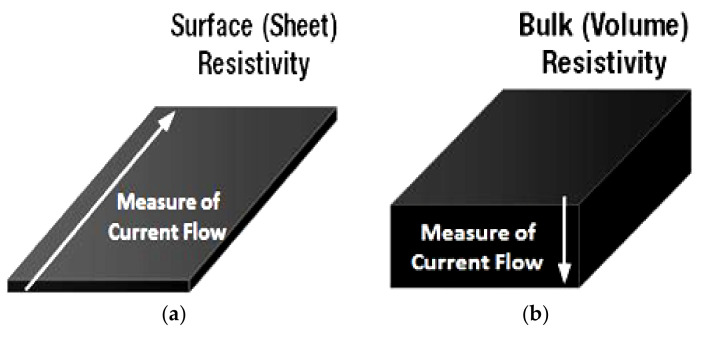
Volume resistivity (**a**) and surface resistivity (**b**).

Table 2 analyzes the main technical characteristics of the material concerning two different Velostat film types, considering that two different base materials can be used with different thicknesses: the electrically conductive EVA copolymer (1801 Sheet Stock) and the polypropylene (1801 Sheet Stock). In particular, the sheet we use belongs to the 1801 stock with a 1/8 inch thickness. Therefore, the “Surface resistivity” and “Volume resistivity” parameters, shown in the following table, represent:

- Surface resistivity ($R_{S}$) (Figure 11a), which measures the electrical conduction of materials with thickness H much less than width W and length L; instead, R is the resistance and ρ the corresponding bulk resistivity of the sample. This quantity is given by:
  (1) $$R_{S}=R\frac{W}{L}=\frac{\rho }{H^{\prime }}$$
  (2) $$R=\rho \frac{L}{{\mathrm{HW}}^{\prime }}$$
- The measurement unit of surface resistivity in the International System is the Ohm (Ω). Besides, it is often used “Ohms per square” (indicated with Ω/sq), dimensionally equal to ohms, but used only for surface resistance to avoid misunderstandings.
- Volume resistivity (Figure 11b) is the current leakage resistance through the insulating material’s volumetric body, expressed in ohm × cm^2^. The higher volume resistivity means lower leakage current and, thus, lower conductance.

In [51], the authors verified the suitability of Velostat material to provide real-time socket pressure profiles. Different bench tests were carried out to determine precision, repeatability, and hysteresis responses of the piezoresistive material. They used a sensor with a sandwich structure consisting of two electrodes and a layer of Velostat material inside with 0.06-mm thickness and 5-mm diameter.

**Figure 12:**
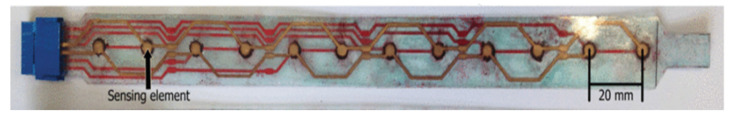
Completed sensor strip featuring twelve sensitive elements, as reported in [51], Copyright 2020 Elsevier_.

The authors realized a sensor matrix with the same configuration using multiplexing for reducing wiring requirements. Figure 12 shows the matrix structure consisting of strips of twelve sensitive elements arranged in sequence along the longitudinal direction. The electrode plates, 2 mm in diameter, were positioned 20 mm apart along the strip and used as contact point for each sensor element. The piezoresistive Velostat film was placed between the twelve pairs of electrodes on the layers of the upper and lower circuits, and the remaining areas of the circuit were isolated from each other with an adhesive layer to avoid shortcircuits. A Bluetooth module was used for data acquisition and transmission.

The authors found slight variations in sensor response on repeated measurements, but they were unable to determine any clear relationship between these variations and the loading speed. They also averaged the loading and unloading responses for twenty loading cycles, comparing the average trends. The results showed that, despite the advantages in terms of low cost, low profile, and ease of integration, Velostat presents numerous shortcomings in terms of accuracy and precision when used as a pressure transducer. The accuracy error ranges between 16% and 48% of the full-scale output, depending on the tested sensor. In addition, thermal response tests indicated a change of the sensor output voltage of up to 67% as the ambient temperature changed.

**Figure 13:**
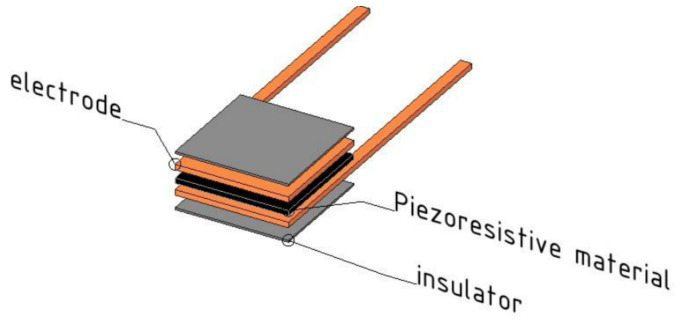
Sandwich-shaped structure for the four sensors realized and characterized in [52].

Further characterization of piezoresistive material was reported in [52]. Four sensors of different materials were realized and tested, based on the Velostat piezoresistive layer and built with the sandwich structure with 1 cm^2^ sensing area, as shown in Figure 13.

Two different tests were performed for each of the four sensors using a universal test machine to determine the following characteristics: the response to various loads, the repeatability of measurements, the hysteresis derived by increasing and decreasing the load, and the ability of sensors to maintain voltage value within acceptable ranges over time without significant drift. The first test evaluated the dynamic response on a range of applied forces. The universal testing machine was programmed to increase load cell strength from 0 to 500 N with a speed of 10 N/s. As soon as the force reached 500 N, the machine kept the load unchanged for 5 s and then started to reduce the force at the same rate. Every 5 ms, the voltage and load data were stored. This procedure was done for the four samples, four times per sample, to determine their repeatability and hysteresis. The second test was performed to assess the stability of the sensors with a constant load over time. During the second test, three different loads (50 N, 150 N, and 400 N) were applied to each sensor for 480 s. The test was performed without removing the previous force, but instead of it, the remaining force was added after each 480 s cycle. The authors demonstrated that the output voltage decreases inversely with the applied load for all tested sensors; particularly, the sensor’s behavior shown a polynomial characteristic ($R=6{.383\times F}^{0.3793})$ as function of the applied force (F), typical of this kind of sensors.

This sensor was characterized by smaller voltage variations (about 0.3 normalized units) within the applied forces’ range from 0 to 500 N; therefore, it has the smallest sensitivity compared with other sensors. However, the hysteresis test demonstrated that the sensor obtained the best results resulting in the lowest error percentage between the increase and decrease of the load. This observation suggests that Velostat or similar materials realized as films surfaced with carbon particles could be used in devices where sensitivity does not affect the specific application, i.e., not wide pressure range. The authors also demonstrated that the sensor made with the Velostat film has the maximum standard deviation, which means that it can be more unstable than the others tested sensors. However, no temperature measurements were made during the test, thus it is not possible to determine whether the environmental parameter affected the results. Moreover, the few test repetitions can help temperature play an even more critical role in obtained results as well as other factors, such as the humidity or contact area.

### 3.2. Structure of the Realized Pressure Sensors, Experimental Setup, and Methodology for the Characterization of the Piezoresistive Sensors

**Figure 14:**
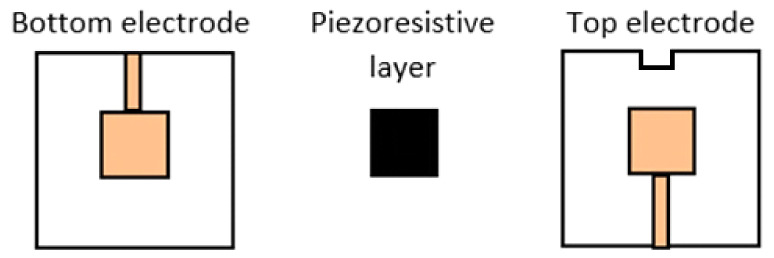
Sandwich configuration used for the realization of the Velostat-based pressure sensors.

**Figure 15:**
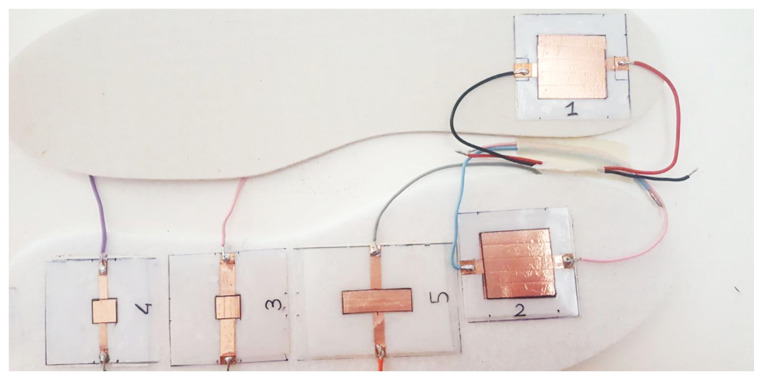
Piezoresistive sensors realized with different sizes and shapes placed on a felt insole.

This section reports the results of the characterizations of several Velostat-based pressure sensors with different shapes and sizes to better understand the material’s behavior. The tested sensors were based on a sandwich structure (Figure 14). The piezoresistive layer was placed between two PVC (i.e., polyvinyl chloride) supports, with 0.2-mm thickness, on which were symmetrically placed two copper electrodes. The Velostat layer, placed between the two electrodes, was not glued to the substrates but sealing the sensing area’s counter by epoxy glue. Furthermore, two copper paths were connected to the electrodes to make them accessible for electrical resistance measurement. In this way, several pressure sensors have been realized with different sizes and shapes (Figure 15).

**Figure 16:**
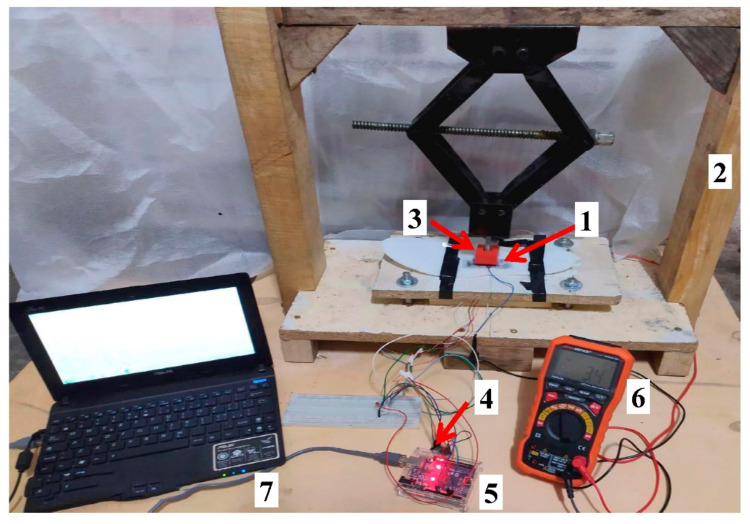
Experimental setup employed for the characterization of the Velostat-based piezoresistive sensors, with highlighted the main sections.

To characterize the piezoresistive sensors, a suitable setup was developed; it is constituted by a self-built press (2 in Figure 16) equipped with a 3D printed adapter with dimensions equal to those of the tested sensors; in this way, the applied force is uniformly distributed on the sensor surface (3 in Figure 16). The press includes a movable plate placed on two flat half-bridge load cells in the lower part, with a capacity of 50 kg (model GML670, manufactured by Gavin Electronics Technology Ltd., Shaanxi, China), connected to obtain a full-bridge Wheatstone bridge configuration. These load cells are featured by 0.05 mV/V comprehensive error, 1 ± 0.1 mV/V output sensitivity, 0.03% FS non-linearity, and 0.03% FS hysteresis. The two load cells were connected to an electronic conditioning and acquisition module based on the IC HX711 (manufactured by Avia Semiconductor, Xiamen, China, 4 in Figure 16), including a 24-bit analog-to-digital converter together with a pre-stage-programmable amplification (PGA-Programmable Gain Amplifier). The load cells’ signal was acquired by a microcontroller board (Arduino UNO, Somerville, MA, USA; 5 in Figure 16) using a two-wire communication interface and displayed on the PC’s serial monitor (7 in Figure 16).

The experimental setup was calibrated using M1 class calibration weights of 1 kg (model WAM1K1, manufactured by Società Bilanciai Porro S.r.l, Milano, Italy) and 20 kg (model WM1NK20, manufactured by Società Bilanciai Porro S.r.l) to determine the gain factor set in the firmware of acquisition section and used to measure the applied load from the signal provided by the load cells. Furthermore, the setup calibration was constantly verified before every measure, applying the 1 kg calibration weight on the press base and checking the rightness of the detected load. Operating in this way, we consider the load measurements provided by the developed setup to be fully reliable.

Besides, the setup includes a digital multimeter (model PM8236, manufactured by Peakmeter^®^, Shenzhen, China; 6 in Figure 16) to measure the electrical resistance of the sensor following the application of pressure (R_pressure_) and the corresponding recovery value (R_recovery_) of the sensor. Preliminarily, the sensors were applied to the support material by double-side tape or epoxy glue so that the insole remains perfectly adherent to the base. In particular, the modifications of sensor response as a function of both support material and fixing method have been analyzed in the following Section 4.1.

The used measurement procedure involved applying a load for 8 min before the resistance measurement to obtain a steady R_pressure_ value; afterwards, the load was removed from the sensor, waiting for 8 min before the resistance measurement to reach a steady R_recovery_. Thus, three different measurement campaigns have been carried out for each sensor averaging the obtained results to derive the average resistance vs. load characteristic. Once the measurements were completed with the first fixing method, the second one was tested, leaving the sensor at rest for a few hours to allow the glue to dry, and the same procedures were repeated.

## 4. Results

This section reports the results of the characterizations of Velostat-based piezoresistive sensors for different supporting materials and fixing methods to determine the most suitable solution for the integration in the sensing matrix. To our knowledge, this is the first characterization of this type reported in the literature for completeness and accuracy. Additionally, Section 4.1 presents a 3 × 1 cm^2^ Velostat sensor, a size selected for the integration inside the sensing matrix, and hysteresis analysis carried out subjecting it to load/unload cycles. The characterization results are widely discussed in Section 5. Section 4.2 describes the structure of the realized piezoresistive sensing matrix and related acquisition and communication section. Finally, Section 4.3 introduces the smart insole’s development for monitoring the plantar pressure distribution and gait parameters, equipped with a piezoelectric harvesting section for scavenging energy from the walking. Then, the device wirelessly transmits the acquired data to a PC or tablet through an integrated BLE module, where these last are stored and displayed through a suitable Processing^®^ application.

### 4.1. Experimental Results Related to the Characterization of the Realized Piezoresistive Sensors

At first, we tested five different piezoresistive sensors, named sensor i (i = 1,2,…5), with different sizes. The sensors have been applied to a felt slab using, at first, the dual-side tape, and then, the epoxy glue to determine the effect of the fixing method on the resistance vs. force sensor characteristics. All sensors were subjected to an ascending force from 0 to 50 kgf and following the procedure previously described.

**Figure 17:**
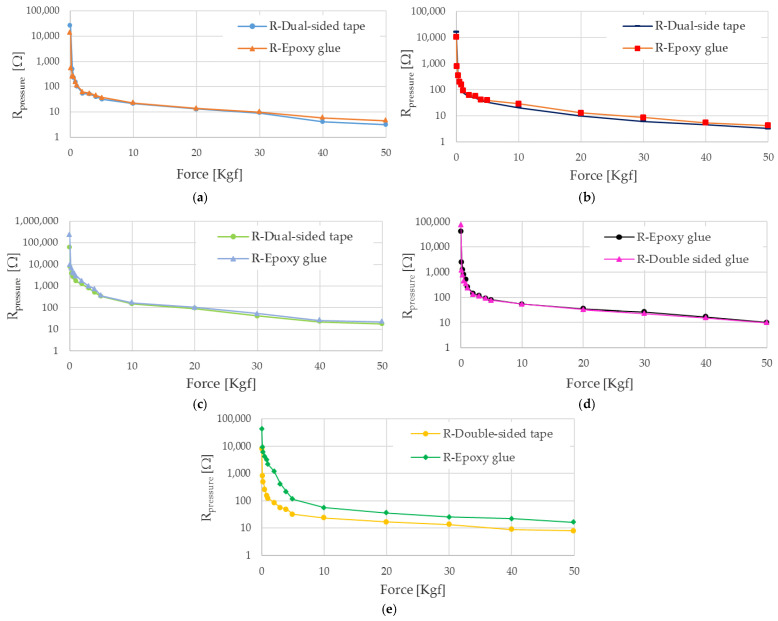
Characteristics R vs. F (expressed in Kgf) for five Velostat-based pressure sensors (logarithmic scale): sensor 1 (3 cm × 3 cm) (**a**); sensor 2 (3 cm × 3 cm) (**b**); sensor 3 (1 cm × 1 cm) (**c**); sensor 4 (1 cm × 1 cm) (**d**); and sensor 5 (3 cm × 1 cm) (**e**).

Specifically, sensor 1 is constituted by a 5 cm × 5 cm PVC support and a 3 cm × 3 cm active area. The R vs. F characteristics are reported in Figure 17a, both when the sensor is applied to the support using double-sided tape and epoxy glue. The sensor was featured by an initial resistance (defined as R_oi_) equal to 31.5 kΩ and 15.5 kΩ, for the two fixing methods, reduced to an average value of the recovery resistance (${\bar{R}}_{\mathrm{recovery}}$) of 25.9 kΩ and 13.5 kΩ, respectively, due to the settlement of the sensitive material. Comparing the $R_{\mathrm{pressure}}$ values for the two fixing methods, the epoxy glue fixing shows a higher resistance value, for a given applied force, than that obtained with the double-sided tape, on average of 33.7%. This effect is due to the sensor stiffening due to the glue layer between the sensor and support. *Sensor 2* has the same dimensions as sensor 1, whose characteristics are depicted in Figure 17b; it is featured by R_oi_ values equal to 16.8 kΩ and 11.3 kΩ, for the dual-sided tape and epoxy glue fixing methods. These values have been reduced to 15.6 kΩ and 10.4 kΩ average ${\bar{R}}_{\mathrm{recovery}}$ values, respectively. From the reported trends, it can be seen that, in the first measurement campaign, carried out by fixing the sensor with the double-sided tape and with the epoxy glue, the resistance trends are almost the same. In contrast, the ${\bar{R}}_{\mathrm{recovery}}$ values have considerable differences, even some kΩ, with greater values for the fixing with double-sided tape. This effect is probably due to better adhesion of the sensor to the slab’s surface for the fixing with the epoxy glue, which induces higher pre-stress on the material, resulting in a lower resistance value. Similarly to sensor 1, the ${\bar{R}}_{\mathrm{pressure}}$ values obtained with double-sided are lower than those resulting from the epoxy glue fixing, on average 46.9%.

Instead, sensor 3 and sensor 4 are constituted by a 3 cm × 3 cm PVC base including a 1 cm × 1 cm Velostat layer. Sensor 3 is featured by a R_oi_ of 235.1 kΩ for the fixing with the double-sided tape, as well as 94.7 kΩ for the epoxy glue; the obtained results indicate a R_oi_ of 230.4 kΩ and 61.3 kΩ for the two considered fixing methods (Figure 17c). From the value reported in Figure 17c, an increase of ${\bar{R}}_{\mathrm{presure}}$, between 5.0% and 43.7%, for the fixing with epoxy glue compared to double-sided tape is evident due to a greater rigidity given to the sensor by the glue layer. Sensor 4 shows a R_oi_ equal to 85.6 kΩ for the double-sided tape and 59.2 kΩ for the epoxy glue (Figure 17d). Similarly, an increase in ${\bar{R}}_{\mathrm{presure}}$ between 1.3% and 49.1% is achieved for the glueing with the epoxy glue, compared to the double-sided tape one. Sensor 5 comprises a 7 cm × 5 cm PVC base, on which are realized two copper contacts and a Velostat layer with 3 cm × 1 cm dimensions. The sensor is characterized by R_oi_ values equal to 63.2 kΩ and 52.5 kΩ for the double-sided tape and epoxy glue fixing. The R vs. F characteristics for the two fixing method are reported in Figure 17e; in this case also, the ${\bar{R}}_{\mathrm{presure}}$ values obtained by fixing the sensor with epoxy glue are greater than those obtained with double-sided tape, between 48.4% and 95.0%.

**Figure 18:**
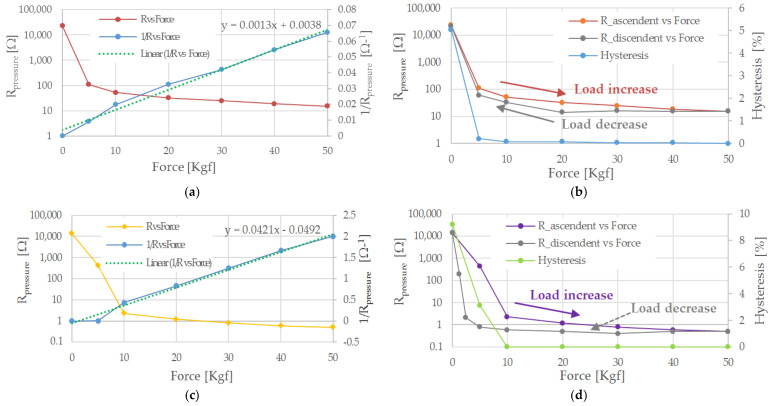

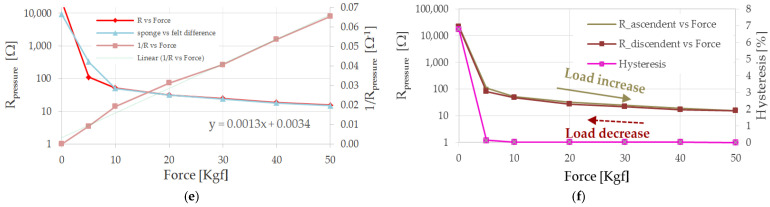
Characteristics of sensor 6 for different support material: rigid support (**a**), sponge (**b**), and felt insoles (**c**). For each support, the trend of ${\bar{R}}_{\mathrm{presure}}$ as a function of the applied force (in logarithmic scale), from 0 Kgf to 50 Kgf, has been determined (**a**,**c**,**e**), along with the inverse of ${\bar{R}}_{\mathrm{presure}}$ besides, the responses of sensor 6 to ascending and descending loads were shown (**b**,**d**,**f**), along with the hysteresis trends.

Moreover, another 3 cm × 1 cm piezoresistive sensor, called sensor 6, has been tested to determine the R vs. F response for different support materials: a rigid base, a sponge slab, and a felt insole. At first, the sensor was placed on the press’s rigid support and changing the applied force between 0 Kgf and 50 Kgf, considering 8 min recovery time between consecutive measurements. These data have been obtained as the average value of the measurements carried out on four different measurement campaigns. The results of this characterization are shown in Figure 18a (red curve), along with the characteristic related to the sensor resistance’s reciprocal as the applied load varies. This observation confirms the previous hypothesis related to the hyperbolic trend of the ${\bar{R}}_{\mathrm{presure}}$. Indeed, the resistance reciprocal (blue curve, Figure 18a) of the sensor is close to a linear trend, featured by an average error of 565 × 10^−6^ Ω^−1^ and a mean square error of 4.26 × 10^−5^ Ω^−1^ from the linear regression (green curve, Figure 18a). Afterwards, sensor 6 has been subjected to loading/unloading cycles from 0 Kgf to 50 Kgf, leaving a rest–pause between consecutive loads of 8 min before to measure the resistance. In Figure 18b are reported the characteristic for ascending (curve orange) and descending load (grey curve); as evident, the sensor shows a hysteresis in the two responses, already observed in previous characterizations [52]. The hysteresis is the maximum difference between the sensor’s responses for a single load once applied in ascending and descending way. The hysteresis error is expressed by the following relation:

(3) $$\mathrm{Hysteresis}\left(\%\right)=\left|\frac{R_{\mathrm{load}}{\;-\;R}_{\mathrm{unload}}}{R_{\max}\;{-\;R}_{\min}}\right|\times 100\%$$

where $R_{\mathrm{load}}$ and $R_{\mathrm{unload}}$ represent the responses in ascending and descending load, respectively, whereas $R_{\max}$ and $R_{\min}$ are the maximum and minimum values of the sensor responses.

Afterwards, sensor 6 has been glued on a sponge insole using epoxy glue and tested according to the modalities previously described. The trend of ${\bar{R}}_{\mathrm{presure}}$ as a function of applied force (yellow curve) and its reciprocal (blue curve) are depicted in Figure 18c. Comparing the graphs of Figure 18a with those in Figure 18c, the effect of the support on the sensor’s response is evident. The higher compressibility of the sponge support causes a greater deformation of the piezoresistive sensor, and thus, a more significant reduction of the sensor’s resistance; therefore, a more rapid reduction of the electrical resistance has been found for a given value of the applied load.

Similarly, the sensor applied to the sponge slab was subjected to a loading/unloading cycle from 0 Kgf to 50 Kgf (Figure 18d). As evident from the following figure, the support greatly affects the sensor hysteresis; this last increased for reduced loads (0–10 kg); this result can be explained by a transient deformation of the slab, leading to a variation of the sensor response in the instants following the load removal.

Subsequently, sensor 6 was glued onto a felt slab using epoxy glue and characterized according to the same methods described above. The characterization results for sensor 6 are shown in Figure 18e, along with the sensor resistance’s reciprocal. Comparing the obtained trend with that determined for the sponge insole, a slight difference between the two characteristics is evident, attributable to the support on which the sensor has been applied. Specifically, the characteristic obtained with the felt insole is slightly higher than the same obtained with the sponge one, probably due to the former’s lower compressibility. The difference between the two characteristics is between 2780 Ω and 0.8 Ω, decreasing monotonously as the applied load increases since the deformations obtained with the two types of support tend to converge (light blue curve in Figure 18e).

Similarly, the sensor placed on the felt insole was subjected to successive loading cycles (from 0 to 50 kg, grey curve in Figure 18f) and unloading (burgundy curve in Figure 18f) to evaluate the hysteresis in the response of the sensor–insole system (fuchsia curve in Figure 18f). As evident from the results shown in the following figure, the sensor’s hysteresis is significantly reduced compared to the similar characteristic obtained with the sponge insole; presumably, this is due to the greater ability of the felt insole to recover its initial shape.

After the static characterizations of the Velostat-based sensor, dynamic tests have been carried out on the eight 3 × 1 cm^2^ sensors included in the assembled sensing matrix. Specifically, a memory effect, not observed on static characterizations, has been observed, thus requiring a firmware compensation, as described in the following Section 4.4.

### 4.2. Structure of the Realized Piezoresistive Sensing Matrix and Related Acquisition Section

This subsection describes and analyzes the development and realization of an insole prototype constituted by a matrix of eight piezoresistive sensors, each based on a Velostat layer. The acquisition section for detecting each sensor’s resistance variations will also be discussed, relying on an analog multiplexer and an Arduino Lilypad acquisition board.

**Figure 19:**
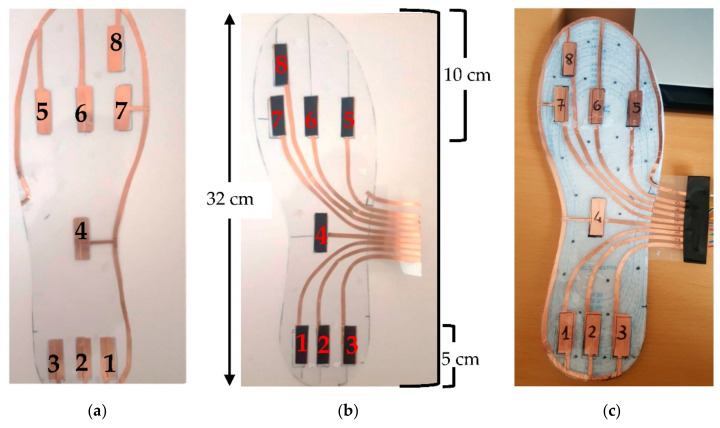
View of the sensing matrix’s prototype consisting of 8 piezoresistive sensors before assembly: top layer (**a**); bottom layer (**b**); and assembled sensing matrix (**c**).

The developed sensing insole is constituted by a matrix of 8 piezoresistive sensors distributed respectively in the toe areas (i.e., the sensors 5, 6, 7, 8), in the central part (sensor 4) and on the heel (sensors 1, 2, 3), as depicted in Figure 19. This distribution was chosen since, from the literature analysis reported in the previous Section 2, such foot areas are more involved in the plantar weight distribution.

The structure of the piezoresistive matrix respects the sandwich structure described in Section 3.2 for the realization of tested FSR sensors; precisely, eight layers of Velostat piezoresistive material, with 3 cm × 1 cm size, have been placed between two layers of PVC on which eight symmetrically couples of copper electrodes are deposited. Next, the piezoresistive layers (black boxes in Figure 19) were fixed at an end to facilitate the alignment of the two PVC layers, and subsequently, the areas around the electrodes were sealed employing epoxy glue. Finally, the bottom electrodes’ connections were created by copper tape and exposed on the side of the insole to make them accessible for the conditioning section. In contrast, all sensors’ top electrodes were connected by a single copper track for simultaneously grounding them, as described (Figure 19c).

**Figure 20:**
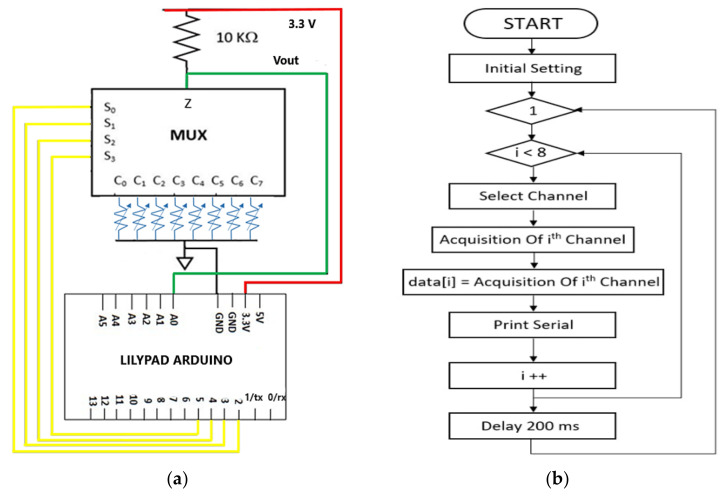
Schematic representation of the acquisition section of the signals related to the piezoresistive sensors of the developed piezoresistive matrix (**a**); flowchart of the Arduino board’s firmware for acquiring the resistance from the eight piezoresistive sensors (**b**).

The proposed acquisition section includes an Arduino Lilypad board and a CD74HC4067 (manufactured by Texas Instruments, Dallas, TX, USA) analog multiplexer/demultiplexer. The proposed architecture, shown in Figure 20a, provides that each sensor of the sensing matrix, represented as variable resistances, is connected with the ground electrode, common to the Arduino board and the other to a multiplexer input from C_0_ to C_7_. Additionally, the single Z output is connected to a high precision pull-up resistor (i.e., 0.1% tolerance) with a nominal value of 10 kΩ (model MRA0207, manufactured by Arcol Ltd., Cornwall, UK), to which a voltage of 3.3 V has been applied via the Arduino board. This last is also used to drive the multiplexer control bits (from S_0_ to S_3_), through four distinct general-purpose input–output (GPIO) pins, from D_2_ to D_5_. The analog voltage V_OUT_, related to a single selected sensor, is acquired using a single analog channel (A_0_) of the Arduino board. According to the previously derived characteristics, this last acquires the resistance data by 10-bit ADC and processes them to extract the pressure data. Besides, the Lilypad board manages the multiplexer enabling/disabling to reduce its power consumption when the stage is not busy in an acquisition.

The CD74HC4067 multiplexer is featured by a non-negligible ON resistance (R_ON_) (R_ON_ = 70 Ω (typical value), R_ON_ = 160 Ω (maximum value), @ Supply Voltage = 4.5 V, I_O_ = 1 mA) for the considered application and also varies with the applied voltage [53]; therefore, the characterization of R_ON_ is needed to compensate for the resistance measurements, avoiding its overestimation. Thus, the R_ON_ was measured by placing a 220 Ω pull-up resistor to the Z pin and grounding the pins from C0 to C7; in this way, on each multiplexer channel, a voltage divider between the common 220 Ω pull-up resistor and the R_ON_ resistance was obtained. Then, the Arduino board was employed to iteratively acquire the voltage on the R_ON_ resistance of each multiplexer channel to calculate the correspondent resistance value. The mean values of the R_ON_ resistance for the eight channels (C0–C7) of the multiplexer, calculated on ten consecutive measurements, are shown in the following Table 3.

As it can be noted, the R_ON_ values vary in the range from 62.80 Ω to 65.31 Ω, thus a mean value equal to 64.25 Ω is considered to eliminate its contribution on the measurement, extracting the exact information on the resistance value of the single piezoresistive sensor, and thus on the applied pressure.

In Figure 20b, the firmware’s flowchart implemented by the Arduino board is illustrated to acquire the resistance values of the eight piezoresistive sensors. As it can be seen, after initial settings, the loop function of the Arduino code, through the for-cycle, iterates, every 200 ms, the acquisition process from the eight piezoresistive sensors. Specifically, each iteration involves the selection of the i-th channel and the acquisition of the corresponding signal, followed by the storage of this information in the i-th element of a storage array (data []), and finally, the serial transmission of the acquired resistance along with cycle counter increase.

Therefore, every 200 ms (working frequency = 5 Hz), all eight channels are acquired, thus a time equal to 25 ms is dedicated for each single-channel (working frequency = 40 Hz). This timing is compatible with the response times of the CD74HC4067 multiplexer; indeed, it has a propagation delay time equal to 15 ns (maximum value, for @Supply Voltage = 4.5 V, Temperature = 25 °C, C_L_ = 50 pF) [53].

### 4.3. Integration of Developed Sensing Matrix and Acquisition Section inside the Smart Insole

**Figure 21:**
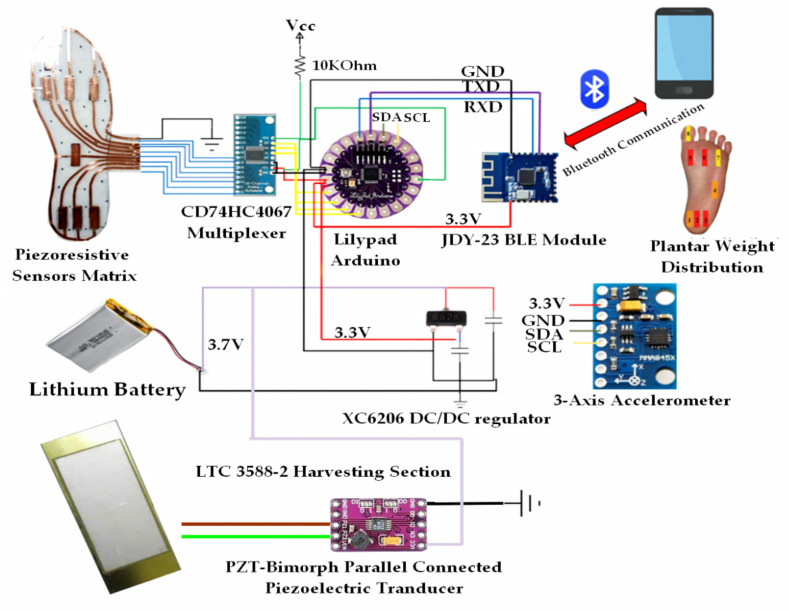
Graphical representation of the architecture of the developed smart insole.

**Figure 22:**
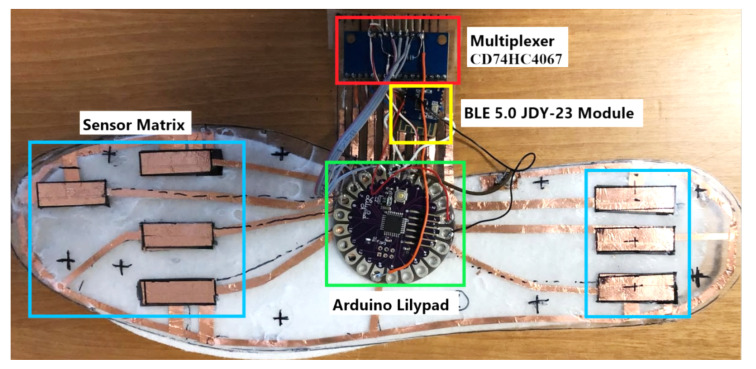
Top view of smart insole prototype constituted by the piezoresistive sensing matrix, acquisition, and communication sections.

The block diagram of the whole smart insole is reported in Figure 21, including the piezoresistive sensing matrix, acquisition and communication sections, and power supply unit. The acquisition section has been assembled on the bottom of the piezoresistive insole (light blue box in Figure 22), which includes the analog multiplexer CD74HC4067 (red box in Figure 22) and the Arduino Lilypad board (green box in Figure 22).

The proposed architecture, described in the previous subsection, provides that each of the eight sensors of the sensing matrix is connected on one side to a ground electrode, common to the Arduino board, on the other side to an input of the multiplexer (called C_0_–C_7_). Moreover, the MUX output is connected to a 10 kΩ pull-up resistor supplied by 3.3 V provided by the power supply unit. The Arduino Lylipad drives the multiplexer’s control bits (from S_0_ to S_3_) through four distinct general-purpose input–output pins (GPIO) and acquires the analog voltage V_OUT_ related to the single selected sensor.

The Lilypad board is also interfaced through the I^2^C (Inter-Integrated Circuit) bus with three axes MMA8452Q (manufactured by NXP Semiconductors, Eindhoven, Netherlands) to count the number of steps performed and determine gait parameters (Figure 21). Particularly, it is performed by extracting the time features of the acceleration trends provided by the accelerometers [45,54]. It is a low-energy three-axis MEMS capacitive accelerometer with 12-bit resolution, suitable embedded functions since featured by reduced dimensions and consumption, and flexible programming options configurable through two interrupt pins. Furthermore, the MMA8452Q sensor is featured by a selectable full-scale value of ±2 g/±4 g/±8 g with optional high-pass data filtering. Moreover, it has a configurable inertial interrupt signal for its awakening from sleep condition to monitor events and remains in a low-power mode during inactivity periods.

The developed firmware for implementing the pedometer relies on comparing the sensor’s total acceleration value with the threshold value set experimentally. If the acceleration is greater than the threshold value, the acceleration sensor increases the step counter and set the event flag to avoid multiple counting of the same step. This flag remains high until the acceleration value falls below the threshold value, indicating that the step is completed. In this case, the acceleration sensor resets the flag and prepares to count a new step as soon as the acceleration value returns to be greater than the threshold value. Therefore, the importance of the threshold value is evident. The optimal value has been determined experimentally testing different acceleration thresholds to ensure correct sensor operation and prevent the overestimation (if the threshold is too low) or underestimation (if the threshold is too high) of the number of steps taken.

**Figure 23:**
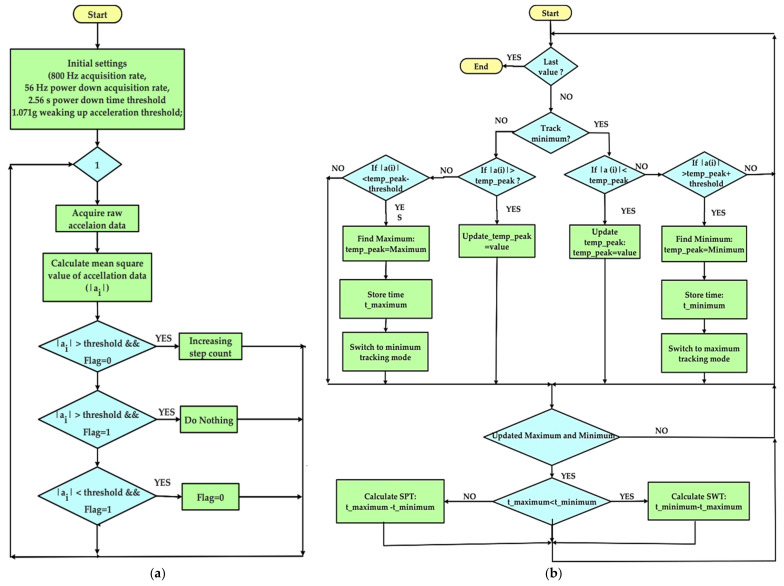
Flowchart related to the pedometer implemented inside the developed smart insole (**a**) and peak detector used to determine the SWT and SPT parameters (**b**).

Furthermore, the embedded auto-weak and sleep to the MMA8452Q have been exploited to reduce the module’s power consumption when the device detects no acceleration. In this condition, the sensor’s data acquisition rate is significantly reduced (i.e., 56 Hz compared to 800 Hz in normal state) when no accelerations are detected for a given time interval (i.e., 2.56 ms) and waking it up, through an interrupt signal, when acceleration exceeds the set threshold is detected (viz 1.071 g). The flowchart related to the implemented pedometer is reported in Figure 23a.

Moreover, to detect these gait events, a peak detection algorithm is implemented (Figure 23b) for determining gait parameters, i.e., the swing time (SWT) and stance phase time (SPT), expressed as:

(4) $$\mathrm{SWT}=\;T_{\mathrm{Toe}-\mathrm{off}}\;{-\;T}_{\mathrm{Heel}-\mathrm{Strike}}$$

(5) $${\mathrm{SPT}=T}_{\mathrm{Heel}-\mathrm{Strike}}\;{-\;T}_{\mathrm{Toe}-\mathrm{Off}}$$

where $T_{\mathrm{Toe}-\mathrm{off}}$ and $T_{\mathrm{Heel}-\mathrm{Strike}}$ are the instants of the toe detachment from the ground and heel support [45,46,55,56]. These parameters, properly monitored, can be used to detect or prevent the onset of any pathologies, such as the formation of diabetes ulcers [39].

Furthermore, the developed smart insole includes a JDY-23 BLE module (manufactured by Shenzhen City Hong Teng Yu Da Electronic Technology Co. Ltd., Shenzhen, China) for transmitting in real-time the acquired data (pressure tuples and gait parameters) towards other smart devices such as a PC, a smartphone or a tablet (yellow box in Figure 22). JDY-23 is a transparent transmission Bluetooth module, since it routes the input data at the output without carrying out any processing operation. It complies with the standard BLE 5.0 protocol and can transmit at a maximum distance of 60 m. The following are the main specifications of the device:

- Transmission power: 4 dB (max)
- Power Supply Voltage 1.8–3.6 V
- Receiving Sensitivity: −97 dBm
- Transmission power: 60 m (max)
- Sizes: 19.6 × 14.94 × 1.8 mm^3^ (l × w × t)
- Bluetooth Version: BLE 5.0 (Compatible with BLE4.0, BLE4.2)
- Awakening Current State: 800 µA (during Transmission)
- Sleep Status of Current Light: <50 µA (Transmission)
- Deep Sleep Current: 9 µA
- Rf-TX/RX peak current: 5 mA

Arduino board wirelessly sends the data to the PC that are displayed by Processing^®^ application and graphically depicted through a color map of the plantar pressure distribution, as described in the following Section 4.4. The insole is powered by a 380-mAh single-cell Li-Po battery (model LW 752035, manufactured by Celewell Technology Co., Hunan, China). For adapting the battery voltage to the 3.3 VDC needed to feed the electronic section included on the insole, a voltage DC/DC regulator (model XC6206, manufactured by Torex Semiconductor, Tokyo, Japan) has been placed (Figure 21). The battery is charged by a piezoelectric harvesting section constituted by a semi-rigid PZT (i.e., Lead Zirconate Titanate) piezoelectric transducer (model KS-70 × 53 × 0.6 mm^3^, manufactured by Dongguan Cosson Electronic Ltd., Shenzhen, China) with bimorph structure and on the electronic section based on the integrated LTC3588-2, produced by Linear Technology and integrated into the insole. Precisely, the transducer was placed in the heel area to take advantage of pressure variations in both the foot’s support and take-off phases. The LTC3588-2 is a fully integrated harvesting section properly designed for alternate sources, like RF and piezoelectric ones. Furthermore, it includes a low-loss rectifier and a high-efficiency buck converter and implements a UVLO mechanism (Under Voltage Lockout) to reduce power consumption when the source does not provide any power.

**Figure 24:**
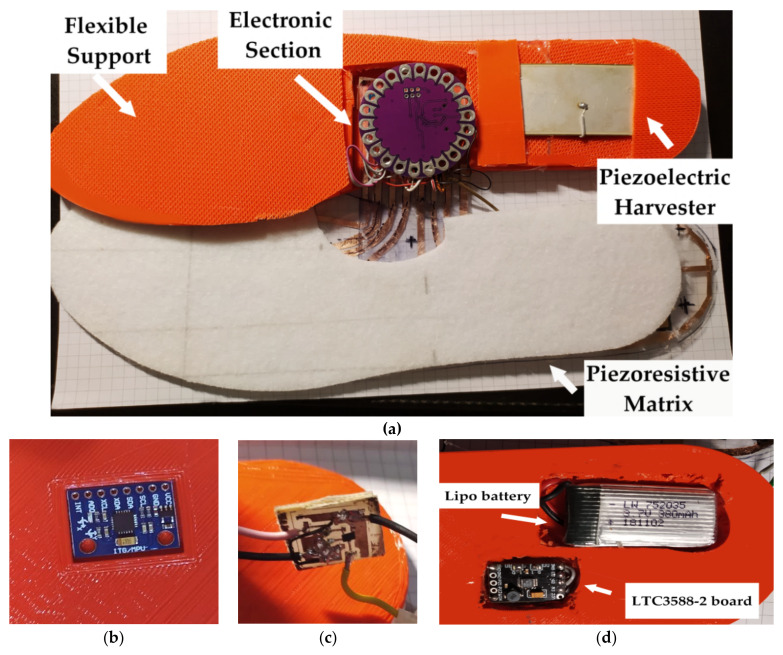
Image of the disassembled slab, which highlights the main sections (**a**) and detail of the housing where the three-axis accelerometer MMA8452Q is placed (**b**) and detail of the voltage regulator used to feed the insole (**c**); insole bottom where are integrated the 380-mAh Lipo battery and the harvesting section based on the LTC3888-2 board (**d**).

The acquisition and communication section was folded and placed into TPU-made (Thermoplastic polyurethane) flexible support (Figure 24a). Next, proper housings have been realized to place the various electronic units of the device (Figure 24b). Finally, the accelerometer has been installed on the insole’s bottom at the forefoot (Figure 24b), as well as the battery, harvesting circuit and DC/DC regulator in correspondence with the heel area (Figure 24c,d), realizing channels inside the insole to hide the connections.

### 4.4. Processing^®^ Application for Data Analysis from the Smart Insole Testing

This section discusses the development of the Processing^®^ code to post-process the acquired pressure data and display them through a color map. Subsequently, the tests on the developed smart insole are reported to validate the sensing matrix implementation.

As previously described, the Lilypad board cyclically connects the eight piezoresistive sensors to a single 10 kΩ pull-up resistor by driving the CD74HC4067 multiplexer, and then, acquires the analog voltage provided by the i-th sensor (Figure 20b). From the gathered voltage values, the resistance of the i-th sensor is calculated to determine the applied pressure, taking into account the R_ON_ mean value of the multiplexer channel (i.e., 64.25 Ω). A linear 1/R_pressure_ vs. pressure characteristic has been supposed (as discussed in Section 5), and the mean slope used to calculate the pressure values; for 3 × 1 cm^2^ sensors, a conversion coefficient equal to $38{.23\;\times \;10}^{-9}\;\Omega /\mathrm{Pa}$ has been used, derived from graphs in Figure 18e. Then, the acquired pressure tuple is transmitted by the onboard BLE module; the acquisition and communication cycle is repeated every 200 ms (5 Hz sampling rate, incrementable up to 480 Hz, limited by the ADC conversion time, i.e., 260 µs).

**Figure 25:**
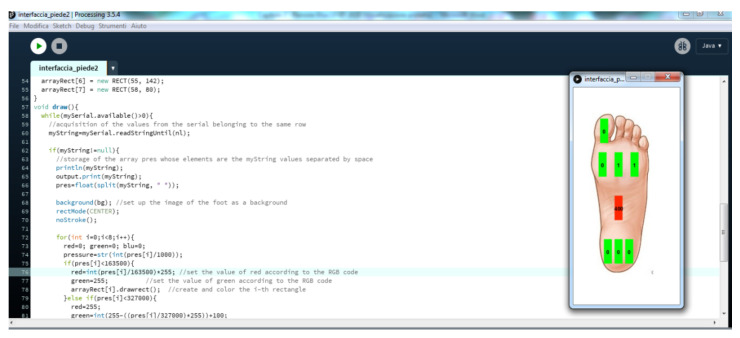
Graphical interface realized with Processing^®^ for displaying the incoming pressure data in the form of a color map.

The host PC receives the acquired data through a virtual serial interface, processes and displays them using a custom Processing^®^ application. This last iteratively reads the incoming data and parses them into an eight elements array, containing the pressure in the different foot sole positions. Then, an array of 8 RECT objects is instantiated, each corresponding to the i-th position on the insole. The firmware evaluates the pressure value acquired from the i-th sensor, determining the filling color of the corresponding rectangle, thus obtaining the color map of the plantar pressure distribution (Figure 25).

To test the insole, a 200-kPa load was applied to each sensor of the sensing matrix using the setup of Figure 16. Table 4 shows the data provided by the smart insole; a good agreement was obtained between the applied pressure and performed measurements, with a percentage error lower than 4.8%. Besides, when a load is applied on a single sensor, the detected pressure of neighboring ones are affected due to local stress.

Based on experimental results, after an instantaneous solicitation, a memory-effect was observed in the following cycle, with a conservation of about 30–40% of the previously detected resistance. This effect tends to disappear after two acquisition cycles; it was compensated in the insole firmware adding a corrective term depending on previous pressure value in the same position ($P_{s,t-1})$ and the time distance from current sample ($P_{s,t})$:

(6) $${P*}_{s,t}{=P\;}_{s,t}{\;-\;C\;\times \;P}_{s,t-1}{\times e}^{-\Delta t/\tau },$$

where ${P*}_{s,t}$ is the adjusted version of the s-th pixel in the time t, C an adjusting coefficient, τ the time constant related to the pressure time variation and Δt the time interval between consecutive samples (i.e., Δt = 200 ms). For the developed firmware, we empirically determined the parameters of the Equation (6), resulting in C=0.97 and τ=223 ms.

**Figure 26:**
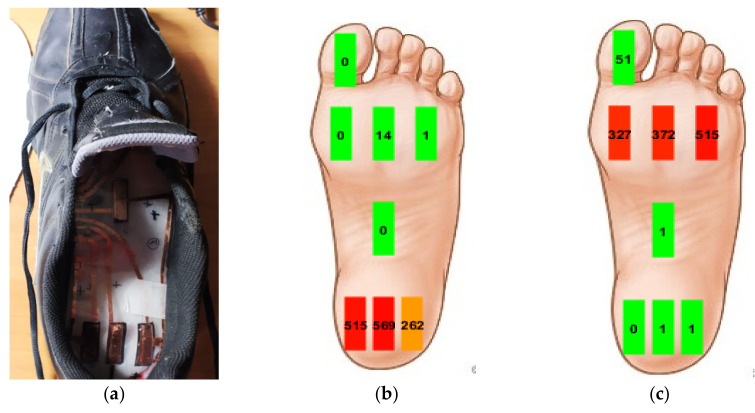
Picture of the piezoresistive insole placed inside a size 45 shoe (**a**); pressure map during the first phase of the step, when the weight is applied on the heel (**b**); second phase of the step with the weight mainly distributed on the forefoot (**c**).

The developed smart insole was inserted inside a size 45 shoe to real-time monitor the plantar pressure distribution during a walk at 1 ms^−1^ speed (Figure 26a). This test allowed to verify the insole correct operation in dynamic condition over 180 s observation interval. In the first phase of the step, when the weight is distributed on the heel (Figure 26b), the sensors 1, 2 and 3 are most stressed; in the second phase, the pressure is exerted on the forefoot, respectively on the sensors 5, 6, 7 (Figure 26c). Sensor 4, placed at the sole center, shows in both cases slight stress due to the natural curvature of the foot sole.

**Figure 27:**
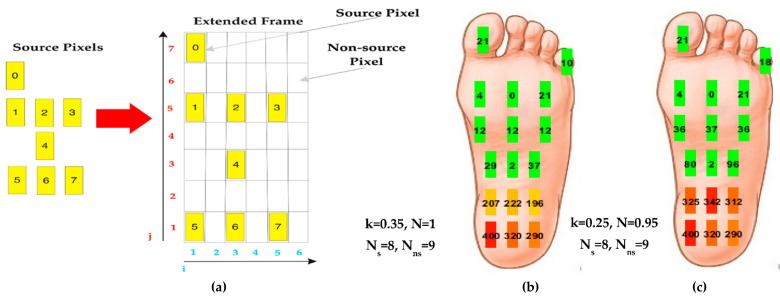
Extended pressure maps by applying the interpolation method proposed in [28] constituted by 17 pixels ($N_{s}{=8,\;N}_{\mathrm{ns}}=9$ (**a**): the first one obtained with k=0.35, N=1 (**b**) and the latter with k=0.25, N=0.95 (**c**).

Subsequently, the plantar pressure map was extended using an interpolation method proposed in [28]. This last evaluates the pressure value in an unknown position, called a non-source pixel, as a weighted linear combination of pressure values in known positions, called source pixels. Specifically, the pressure value in an (i,j)-th non-source pixel ($P_{\mathrm{ns}}\left(i,j\right)$, Figure 27a) is obtained by summing the s-th source pixel pressure values ($P_{s}$) weighted for terms inversely proportional to the distance between them ($d_{\mathrm{ijs}})$.

(7) $$P_{\mathrm{ns}}\left(i,j\right)=N\times \left[\overset{N_{s}}{\underset{s=0}{\sum }}P_{s}{\times e}^{{-k\times d}_{\mathrm{ijs}}}\right],$$

where N is a normalizing factor, $N_{s}$ and $N_{\mathrm{ns}},$ the number of source and non-source pixels, and k, a parameter regulating the influence of source pixel pressures on the non-source ones.

This interpolation method was implemented into the Processing^®^ application to extend the acquired pressure frame, constituted by 8 source pixels ($N_{s}=8$) provided by the insole into an extended frame with 17 pixels. Figure 27b,c shows two extended pressure maps, obtained with different parameters; the first one was obtained setting the k coupling parameter to 0.35 and the N normalizing factor to 1 (Figure 27b), whereas the latter was for k and N equal to 0.25 and 0.95, respectively (Figure 27c). Comparing the two maps, the parameter effect is evident; when increasing k, the impact of the neighbouring pressure values of source pixels is reduced. These two couple of values were the most performant between several considered parameter combinations.

## 5. Discussion

**Figure 28:**
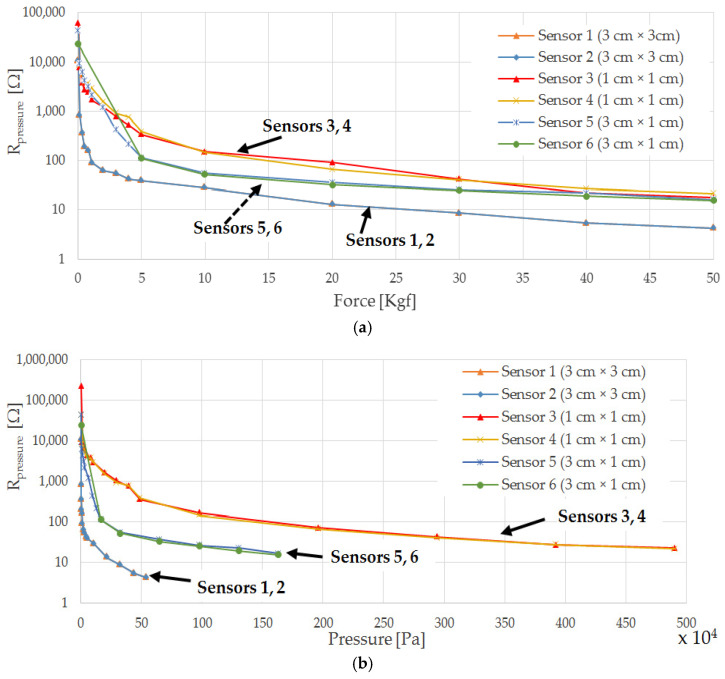
Summarizing graphs of characteristics (${\bar{R}}_{\mathrm{pressure}}$ as a function of applied force (in Kgf) (**a**) and pressure (in Pa) (**b**) for previously tested sensors, all applied by epoxy glue on a felt base.

After the characterizations carried out on the different Velostat-based sensors, reported in Section 4.1, a comparative analysis is presented to determine the most suitable structure and sizes to be used for the sensing insole. Figure 28 shows the characteristics of sensors from 1 to 6 as a function of the applied force and pressure, when fixed on a felt slab using epoxy glue. As evident, the six sensors have similar characteristics, with a hyperbolic trend as the pressure varies.

The sensor resistance is given by the sum of the volume resistance of the piezoresistive material ($R=\frac{\rho L}{A}$, with ρ the bulk resistivity, L the length and A the cross-section) and the contact resistances between the Velostat layer and copper electrodes depending on the applied force ($R\propto \frac{\rho \;K}{F}$, with ρ the bulk resistivity, F the applied normal force, and K parameter depending on elastic properties and roughness of the sample [57]). As the applied force increases, the bulk resistance and contact resistances decrease due to the reduced piezoresistive layer thickness and the increased contact area at a microscopic level between copper electrodes and Velostat layer. Comparing the obtained characteristics, it is evident that sensors 1 and 2, having the larger active area (3 × 3 cm^2^), are featured by a lower resistance value for a given applied pressure (blue and orange curves in Figure 28b), due to lower values of the Velostat layer’s bulk resistance and contact resistances [58]. Similarly, sensors 3 and 4, having a smaller area (1 × 1 cm^2^) than sensors 1, 2 and 5, 6 (9 and 3 times, respectively), present higher bulk resistance and contact resistance values for a given applied pressure.

**Figure 29:**
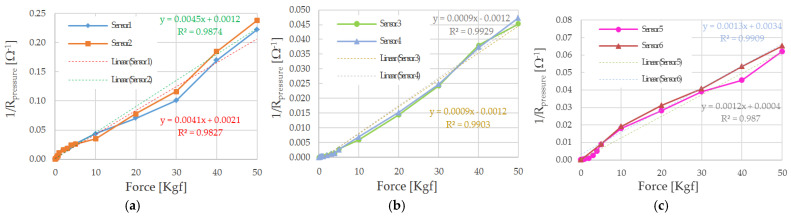
Trends of the reciprocal of sensor resistance as a function of the applied force (expressed in Kgf) for sensors 1 and 2 (3 cm × 3 cm) (**a**), sensor 3 and 4 (1 cm × 1 cm) (**b**), and sensor 5 and 6 (3 cm × 1 cm) (**c**).

In [59], the authors reported a hyperbolic trend of Velostat piezoresistive film’s responses as a function of the applied force. Figure 29 depicts the obtained trends of resistance reciprocal (${\bar{R}}_{\mathrm{pressure}}$) as a function of the applied load for all the sensors described and tested in Section 4.1. As it can be noticed, the obtained characteristics are very close to linear regressions with determination coefficients (R^2^) between 0.9827 and 0.9929.

Table 5 summarizes the slopes of the reciprocal resistance trends for the six tested sensors. Sensors 1 and 2 are featured by higher slope due to the larger area; however, their aspect ratio poorly adapted to the sensing matrix structure and the number of monitored points. For these reasons, 3 × 1 cm^2^ sensors have been chosen to realize the sensing matrix given a more suitable aspect ratio and good slope values.

The developed smart insole is featured by a peak power consumption of 33.75 mW in active mode as well as only 5.81 mW in sleep mode; in fact, the system heavily exploits the low-power modalities of employed components. During the tests, the insole was programmed to perform ten acquisitions of the pressure values and gait parameters per minute. After each acquisition, the Arduino board brought in low-power mode the BLE module and the analog multiplexer, as well as enabled the auto-weak/sleep mode of the MMA8452Q accelerometer. Then, the Atmega328p microcontroller of Arduino Lilypad board disabled the unnecessary components, like ADC, BOD (Brown Out Detector), and timers, reducing its power consumption to about 1.74 mA (for 8 MHz clock signal), but guaranteeing the acceleration monitoring. These current values were measured by a bench multimeter (model GDM-8351, manufactured by Gwinstek, Taipei, Taiwan), acting as an ammeter, in series to the system supply line and setting 1 kHz sampling rate.

Since the time duration of the acquisition and transmission phase was equal to about 6 ms, the charge required for each hour of the system operation was 1.76 mAh. Therefore, the device autonomy in absence of any energy contribution from the piezoelectric harvesting section and supposing a 40% discharge margin of the Lipo battery is given by (8):

(8) $$\mathrm{Autonomy}=\frac{\mathrm{Battery}\;\mathrm{Capacity}\times (1\;-\;\mathrm{Discharge}\;\mathrm{Margin})}{\mathrm{Charge}\;\mathrm{Consumption}\;\mathrm{per}\;\mathrm{hour}}=\frac{380\;\mathrm{mAh}\times \left(1\;-\;0.4\right)}{\;1.76\;\mathrm{mAh}/h},\;=129.54\;h\;(9.29\;\mathrm{days}\;\mathrm{for}\;14\;h\;\mathrm{daily}\;\mathrm{usage})$$

Therefore, an energy autonomy of 130 h was obtained without any contribution from the harvesting section; supposing a daily use of 14 h, about nine days of autonomy are ensured. As reported in Section 4.3, the insole integrates a harvesting section, constituted by a bimorph PZT transducer placed under the heel, with a conditioning section based on LTC3588-2 IC (Integrated Circuit). The harvesting section has been tested by measuring the scavenged power during normal walking using a data logger (model PM8236, manufactured by Peakmeter^®^, Shenzhen, China) to detect the output current at different walking speeds, measured by GPS Speedometer mobile app (developed by Smart Mobile Tools, Ha Noi city, Vietnam). The extracted power values are summarized in Table 6 for 1 ms^−1^, 1.5 ms^−1^, 2 ms^−1^, and 3 ms^−1^ walking speeds.

The harvesting section can ensure the mean power consumption required by the developed electronic load ($\bar{P}=5.84\;\mathrm{mW}$) for a walking speed higher than 1 ms^−1^ (moderate gait), guaranteeing a small charge surplus for recharging the battery. Otherwise, the device’s energy requirements during the short time intervals in active mode are covered by the 380-mAh Lipo battery.

## 6. Conclusions

Modern technologies are increasingly supporting medical staff and users to monitor the onset or evolution of pathologies as well as gather information related to sport performances. In this paper, the design and realization of a novel smart insole are presented to monitor the plantar pressure distribution and gait parameters (such as SWT and SPT). Particularly, the device includes a low cost and reliable sensing matrix constituted by eight piezoresistive sensors based on the Velostat layer for transducing the pressure applied by the human body into an electric signal. An accurate and complete characterization of Velostat-based piezoresistive sensors has been carried out for different sizes, support materials, fixing methods, and applied pressure trends, demonstrating the suitability of the used sandwich structure for the considered application. Furthermore, a low power conditioning and processing section has been developed and integrated on the smart insole to acquire and process the data from the piezoresistive matrix and an onboard 3-axis accelerometer for detecting the plantar pressure distribution and gait parameters. Moreover, the device integrates an ultra low power BLE 5.0 module for wirelessly transmitting the acquired data toward a PC, tablet, or smartphone, where a custom Processing^®^ application allows to display and process them. In particular, an interpolation method has been implemented for deriving an extended pressure map, enabling a more detailed understanding of the plantar pressure distribution.

Moreover, the developed smart insole is equipped with a piezoelectric harvesting section based on a bimorph PZT harvester and an integrated conditioning circuit (based on the LTC5388-2 IC). It allows scavenging energy from the pressure variations related to the walking and storing it into a 380-mAh Lipo battery used to feed the integrated electronic sections. The onfield tests demonstrated that for a walking speed higher than 1 ms^−1^, the harvesting section could fully cover the energy requirement of the developed device (i.e., $\bar{P}=5.84\;\mathrm{mW}$). However, the integrated Lipo battery can ensure a long lifetime (i.e., 9.29 days for 14 h daily usage) to the device in the total absence of any energetic contribution from the harvesting section.

## Figures and Tables

**Table 1:** Summarizing table with compared smart insoles previously discussed in terms of typology and number of pressure sensors, the availability of wireless connectivity, and acquired parameters.

Work	Pressure Sensors	Number of Pressure Sensors	Availability of Wireless Connectivity	Acquired Data
M. Tahir et al. [34]	Piezoresistive sensors (FSR 402 *Interlink Electronics*)	16	BLE	vGRF
K. Ivanov et al. [35]	Piezoresistive sensors (FlexiForce A301, Tekscan)	9	BLE	Plantar pressure map
Pedar X^®^ [48]	Piezoresistive sensors	99	No	Maximum pressure map Mean value map
SurroSense Rx^®^ [39]	Piezoelectric sensors	8	BLE	Plantar pressure map
A.M. Reyzelman et al. [41]	-	-	BLE	Foot temperature
Y. Charlon [45]	Piezoresistive sensors FlexiForce A401, Tekscan)	1	802.15.4 radio module	Step number Distance covered Gait speed
Y. S. Mustufa et al. [47]	Capacitive pressure sensors	32	Bluetooth	Plantar pressure map Information on user movement and position
G. Rescio et al. [49]	Piezoresistive sensors (CP151 IEE Innovations)	8	No	Plantar pressure map Foot temperature

**Table 2:** Technical characteristics of 1801 Sheet Stock and 1801 Sheet Stock of Velostat film.

Technical Features	Velostat Film 1801 Sheet Stock	Velostat Film 1840 Sheet Stock
Dimensions	11″ × 11″ (280 mm × 280 mm)	
Weight	18.66 g	
Temperature Limits	−45 °C to 65 °C (−50 °F to 150 °F)	
Heat Sealable	Yes	
Volume Resistivity	<500 ohm-cm	
Surface Resistivity	<31,000 ohms/sq.cm	
Hardness	58–62 Shore D	67–71 Shore D
Heat Deflection Temp	38–43 °C @ 264 PSI	100 °C @ 66PSI 50 °C @ 264 PSI
Water Absorption	0.1–0.2%	0.1–0.2%
Vicat Softening	88–92 °C	148 °C
Flammability	4.5–5.5 cm/min	2 cm/min
Impact Resistance	2.9–3.7 ft.-lbs./in. @ 72 °F	8–10 ft.-lbs./in. @ 72 °F
Notched Izod	0.6–1.3 ft.-lbs./in. @ 25 °F	7–9 ft.-lbs./in. @ 25 °F
Maximum Temp. Exposure	150 °F	180 °F
Tensile Strength	1700–2000 PSI	2800–3000 PSI
Flex Modulus	40,000–50,000 PSI	130,000–150,000 PSI
Mold Shrinkage	15–20 mil/in.	10–20 mil/in.
Volume Conductive	<500 ohms-cm	<500 ohms-cm

**Table 3:** Table with reported results of the characterization of R_ON_ resistance of eight channels of the CD74HC4067 multiplexer.

Multiplexer Channel								
	**0**	**1**	**2**	**3**	**4**	**5**	**6**	**7**
$\bar{R_{ON}}\left[\Omega \right]$	65.31	63.19	65.02	63.37	64.70	62.80	64.88	64.80

**Table 4:** Carried out tests applying 200-kPa load on one sensor at a time of the sensing matrix.

	Pressure Value Related to the Sensors’ Insole [kPa]							
Pressed sensor	P_1_	P_2_	P_3_	P_4_	P_5_	P_6_	P_7_	P_8_
No sensor	0.26	0.25	0.16	0.31	0.13	0.79	0.23	0.77
Sensor 1	207.60	9.69	2.09	1.25	2.01	2.85	1.75	0.51
Sensor 2	66.62	209.60	9.02	0.95	1.71	2.01	0.59	0.47
Sensor 3	3.20	0.05	205.15	0.84	2.16	2.14	0.32	0.51
Sensor 4	0.87	0.76	3.23	206.16	1.97	1.54	0.32	0.87
Sensor 5	0.11	0.81	0.71	1.91	201.28	3.22	0.95	0.40
Sensor 6	0.08	0.78	0.63	1.25	3.54	201.43	6.00	0.06
Sensor 7	0.11	0.77	0.71	0.93	0.64	4.04	202.24	0.06
Sensor 8	0.16	0.83	0.89	1.17	1.66	4.18	3.80	209.71

**Table 5:** Summarizing table with slopes of the reciprocal resistance trends for the six tested sensors.

Sensor	Slope [Ω^−1^/Kgf]	Slope [Ω^−1^/Pa]
1 (3 × 3 cm^2^)	0.0041	3.76 10^−7^
2 (3 × 3 cm^2^)	0.0045	4.12 10^−7^
3 (1 × 1 cm^2^)	0.0009	9.18 10^−9^
4 (1 × 1 cm^2^)	0.0009	9.18 10^−9^
5 (3 × 1 cm^2^)	0.0012	3.67 10^−8^
6 (3 × 1 cm^2^)	0.0013	3.97 10^−8^

**Table 6:** Power values provided by piezoelectric harvesting section for different walking speeds.

Walking Speed [m/s]	Output Power [mW]
1	5.97
1.5	6.89
2	7.13
3	8.65

## Data Availability

Data of our study are available upon request.
